# Vascular proteomics in metabolic and cardiovascular diseases

**DOI:** 10.1111/joim.12486

**Published:** 2016-03-04

**Authors:** M. Lynch, J. Barallobre‐Barreiro, M. Jahangiri, M. Mayr

**Affiliations:** ^1^ King's British Heart Foundation CentreKing's College LondonLondonUK; ^2^ St George's HospitalNHS TrustLondonUK

**Keywords:** apolipoproteins, atherosclerosis, diabetes, proteomics, restenosis, vascular biology

## Abstract

The vasculature is essential for proper organ function. Many pathologies are directly and indirectly related to vascular dysfunction, which causes significant morbidity and mortality. A common pathophysiological feature of diseased vessels is extracellular matrix (ECM) remodelling. Analysing the protein composition of the ECM by conventional antibody‐based techniques is challenging; alternative splicing or post‐translational modifications, such as glycosylation, can mask epitopes required for antibody recognition. By contrast, proteomic analysis by mass spectrometry enables the study of proteins without the constraints of antibodies. Recent advances in proteomic techniques make it feasible to characterize the composition of the vascular ECM and its remodelling in disease. These developments may lead to the discovery of novel prognostic and diagnostic markers. Thus, proteomics holds potential for identifying ECM signatures to monitor vascular disease processes. Furthermore, a better understanding of the ECM remodelling processes in the vasculature might make ECM‐associated proteins more attractive targets for drug discovery efforts. In this review, we will summarize the role of the ECM in the vasculature. Then, we will describe the challenges associated with studying the intricate network of ECM proteins and the current proteomic strategies to analyse the vascular ECM in metabolic and cardiovascular diseases.

## Introduction

Vascular smooth muscle cells (VSMCs) synthesize and maintain a complex meshwork of extracellular matrix (ECM) 1. Constituting around 300 proteins, the ECM is an intricate three‐dimensional, noncellular lattice composed of elastin, collagen, proteoglycans (PGs) and glycoproteins 2. The function of the ECM goes beyond providing a supporting framework for the cellular constituents. The ECM is increasingly being recognized as a dynamic structure with multifaceted functions. In addition to providing physical support for tissue integrity and elasticity, the ECM serves as a reservoir for bioactive molecules, including growth factors, cytokines and proteases, that are required for tissue homeostasis 3. The importance of the ECM is demonstrated by the substantial number of pathologies related to ECM abnormalities 4. Of more relevance, ECM dysregulation has been implicated in diseases affecting the vasculature. These include but are not limited to aneurysms 5, vein graft failure 6, varicose veins 7, diabetic vasculopathy 8, restenosis and atherosclerosis 9.

## The ECM of the vasculature

The vascular ECM can be divided into two lower‐order structures: the basement membrane (BM) and the interstitial matrix.

The BM is a specialized ECM protein complex separating the endothelium from the underlying connective tissue 10. The close proximity to the cells provides mechanical stability and controls cell organization and polarity. Some of the main protein components of the BM include laminins; collagens IV, XV and XVIII; perlecan; agrin; and nidogen 11. Perlecan is a particularly large PG (469 kDa) and is essential for the stability of the BM. It has domains for interaction with other components of the BM such as collagen IV and laminins 12. The N‐terminal domain contains the majority of anchoring sites for heparan sulphate PGs, which combined with their position in the BM enable perlecan to participate in signalling events 12. It is interesting that endorepellin, an N‐terminal peptide from perlecan released by proteolytic action, has important anti‐angiogenic properties 13.

The interstitial matrix determines most of the mechanical properties of the tissue 14. Although anchored to the BM, it forms a distinct functional unit that provides the means (via an extracellular compartment) through which signals are distributed and nutrients and fibroblasts migrate 15. The main components of the interstitial matrix in the vessel wall are elastin, collagens I, III, VI and XII and PGs 16.

### Collagen

Collagens are a large family of proteins with triple helix conformations and are present in many tissues. Structurally, the collagens contain three alpha polypeptide chains that form a right‐handed coil with hydrogen bonds linking adjacent chains 17. Heterotrimeric forms in the vasculature include collagens I, IV, V and VI, whilst the homotrimeric structure (with three identical chains) includes for example collagens III, XVIII and XV. The extensive number of collagen subtypes gives rise to an equally extensive variety of functions, ranging from the structural support of tissues to cell adhesion and migration, angiogenesis and tissue repair. The fibrillar forms of collagen are the main tensile element in many tissues. In the vasculature, fibrillar collagens are mainly represented by types I, III and V 18. By contrast, other collagen types form networks (i.e. collagens IV and VIII), which are key components of the BM. Additional forms of collagen are important for cell adhesion and organization (i.e. collagens VI and XIV) and anchoring of other components within the ECM (i.e. collagen VII). Furthermore, some collagens have been found to contain domains that exert regulatory roles when released by proteolytic activity. This includes, for example, endostatin, a C‐terminal fragment of collagen XVIII. Additionally, new forms of collagens have been identified with transmembrane domains, further adding to the ubiquity and multifunctionality of this family of proteins. The detailed classification of collagens has been extensively reviewed elsewhere 19.

### Elastin

Elastin is the principal component of elastin lamellae, the fenestrated layers present in the tunica media of arteries. Similar to fibrillar collagens, the functional form of elastin is a cross‐linked amalgamate of tropoelastins, that is the *elastin* gene product. Elastic recoil imparted by elastic fibres is an essential property for normal cardiovascular function 16. Briefly, large elastic arteries allow blood pressure to be maintained throughout the cardiac cycle by storing a portion of the stroke volume with each systole. Subsequently, discharging that volume with diastole reduces the left ventricular afterload 20. This phenomenon is known as the ‘Windkessel’ effect. Less compliant elastic arteries result in a diminution of the Windkessel effect with age and lead to hypertension, predisposing to conditions such as myocardial infarction, stroke and heart failure.

### Glycoproteins

Glycosylation, the covalent attachment of carbohydrate molecules to a core protein, is a common post‐translational modification (PTM) of ECM proteins. There are two types of glycoproteins: N‐glycosylated glycoproteins, in which the sugar is attached to the amide nitrogen of asparagine, and O‐glycosylated glycoproteins, in which the sugar is attached to an oxygen atom of hydroxylysine, hydroxyproline (Hyp), serine or threonine 21. Due to the presence of specialized domains and carbohydrate residues, glycoproteins are essential components for cell adhesion and for mediating attachment of cells to the BM 21. Laminins and fibronectin are the two most important examples of this class of glycoprotein.

### Proteoglycans

Originally identified as major components of cartilage, PGs were for years thought to be specific for cartilage 22. However, they have since been found in virtually all tissues, where they are distributed within the interstitial matrix and BM. PGs have a basic structure comprising a core protein and a variable number of glycosaminoglycan (GAG) chains usually attached through O‐glycosidic linkages to serine residues in the core protein. One classification system of PGs is based on the type of attached GAGs: (i) chondroitin sulphate and dermatan sulphate, consisting of repeating disaccharide units of galactosamine and either glucuronic acid or iduronic acid, (ii) heparan sulphate, consisting of repeating disaccharide units of glucosamine and either glucuronic acid or iduronic acid, and (iii) keratan sulphate, consisting of repeating disaccharide units of glucosamine and galactose 23.

Within the vessel wall, the two most abundant PGs are large aggregating PGs and small leucine‐rich PGs (SLRPs).

Versican is an important large aggregating PG in the vessel wall, not least due to its involvement in the retention of lipoproteins and development of atherosclerosis (reviewed by Wight and Merrilees 24). Versican has also been implicated in smooth muscle cell proliferation and migration contributing to restenosis 25. As a chondroitin sulphate PG, versican is secreted with a structure consisting of a large globular domain at each end and an intermediate domain carrying chondroitin sulphate chains. Through its globular domains, versican interacts with hyaluronic acid. Versican has high homology at the globular domains to other PGs that bind hyaluronic acid (‘hyalecticans’ such as aggrecan, brevican and neurican), but the intermediate region varies considerably in terms of sequence, length and number of chondroitin sulphate chains. Splice variants, truncated glycoforms and stable degradation products further increase the diversity of this PG family 26.

Small leucine‐rich PGs are a family of biologically active ECM components belonging to the leucine‐rich repeat superfamily of proteins 27. Their dominant feature is the presence of leucine‐rich repeats flanked by cysteine clusters in the core protein. After their synthesis, SLRPs are secreted into the pericellular space where they interact with different extracellular molecules and the plasma membrane, modulating a variety of processes including collagen fibrillogenesis and TGFβ sequestration 28, 29. Prominent vascular SLRPs include decorin, biglycan and lumican 30. Studies in knockout mice have shown some degree of functional overlap between different SLRPs 31.

There are several families of PGs in which the protein core is partly integrated into the plasma membrane, with their GAG‐containing regions exposed on the extracellular side of the membrane. These PGs are important for anchoring and signalling between cells and the ECM. Syndecans and dystroglycans are part of this group and mediate cell–matrix interactions.

## PTMs of the ECM

Extracellular matrix proteins are known to undergo extensive PTMs such as hydroxylation and glycosylation.

### Hydroxylation

As mentioned above, all collagens form triple helices and this characteristic increases molecular stability and provides resistance to tensile stress, which is particularly important for fibril‐forming collagens. Different forms of collagen exist in the vasculature, but in general, a common pattern can be observed for amino acid sequences of every type of collagen; each chain has enriched triplet repeats, starting with a glycine residue followed by proline and Hyp. The presence of this repetitive sequence is key for the constitution of the right‐handed helix, and Hyps provide the substrate for the formation of hydrogen bonds between the adjacent alpha chains. Moreover, this putative triplet is modified in different forms of collagen, resulting in multiple permutations that are responsible for the functional and spatial diversity of the collagen family 32. During collagen synthesis, Hyp is derived from proline oxidation and the modification is introduced at specific positions once the protein has been synthesized. Then, collagens are secreted into the extracellular space. Prolyl‐4‐hydroxylase and prolyl‐3‐hydroxylase catalyse the hydroxylation of specific proline residues in the lumen of the endoplasmic reticulum. The former enzyme reacts on proline within a motif of X‐Pro‐Gly (where X is any amino acid), and the latter appears to require a Pro‐4‐Hyp‐Gly sequence 33, 34.

### Glycosylation

Glycosylation is important for correct folding and stability whilst also contributing to the biological functions of glycoproteins 21. ECM proteins tend to be extensively modified by addition of N‐ and O‐linked large, repetitive GAGs and shorter and diverse N‐ and O‐linked oligosaccharides. N‐linked glycosylation occurs at asparagine within a consensus sequence of Asn‐X‐Ser/Thr (N‐X‐S/T), where X can be any amino acid except proline 35. UniProtKB contains 15,828 sites for the human proteome, where the N‐X‐S/T sequon is annotated as the N‐glycosylation site. It has been suggested that only 27% of all N‐X‐S/T sequons are glycosylated 36, highlighting the importance of studying glycosylation to better understand the properties of the ECM. Unlike N‐glycosylation, there is no consensus sequence for O‐linked glycosylation, which occurs on the amino acids serine and threonine.

## Degradation of the vascular ECM

All vascular cells have the ability to remodel their surrounding ECM. The ECM is subject to the action of proteolytic enzymes, which maintain a dynamic equilibrium between protein synthesis and degradation. Amongst these, enzymes are the members of a disintegrin and metalloproteinase (ADAM) and a disintegrin and metalloproteinase with thrombospondin motifs (ADAMTS) families, serine proteases (e.g. plasmin, neutrophil‐derived elastase and cathepsin G), cysteine proteases (cathepsins B, L and S), aspartyl proteases (cathepsin D) and matrix metalloproteinases (MMPs).

### Matrix metalloproteinases

Matrix metalloproteinases are a family of 23 proteases that use zinc as a cofactor. In general, they are synthesized as inactive pro‐MMPs in which the catalytic domain remains inaccessible due to the interaction of the N‐terminal propeptide with Zn^2+^. Once the propeptide is released by proteolytic action, the catalytic domain is exposed and the enzyme becomes active. After being secreted, pro‐MMPs bind to different molecules of the ECM and lie dormant, forming a reservoir that can be activated immediately upon demand 37. Due to the effectiveness of these enzymes, establishing strict control of their activity is essential. The tissue inhibitors of matrix metalloproteinases (TIMPs) are low molecular weight proteins that bind avidly to MMPs, inhibiting their activity 38. TIMP‐1, TIMP‐2 and TIMP‐3 collectively inhibit all metalloproteinases with different substrate specificity 1. MMP and TIMP activities with respect to cardiovascular disease (CVD) have attracted most attention so far, and although inhibitory antibodies of MMPs have shown effects in animal models, there is significant disparity when tested in patients 15.

### ADAMs and ADAMTSs

The ADAMs family, which is related to the MMPs, can be categorized as either membrane‐anchored ADAMs or as secreted ADAMTSs 39. Given their greater propensity to interact with the ECM, we will primarily focus on the ADAMTS family.

The ADAMTSs are synthesized as inactive pre/pro‐enzymes and contain, like MMPs, a metalloproteinases catalytic domain. The enzyme furin has been implicated in pro‐protein processing and subsequent activation. However, other enzymes may be involved and there is evidence to suggest that ADAMTS‐7 40 and ADAMTS‐13 41 are catalytically active with their pro‐domain attached. The major ECM‐related functions of the ADAMTS family appear to be the cleavage of the large aggregating PGs (aggrecan and versican) as well as the processing of pro‐collagen to collagen via removal of the N‐terminal pro‐peptides 42.

Seminal work by Jonsson‐Rylander *et al*. 43 indicated a role for ADAMTS‐1 in atherosclerosis. The authors hypothesized that the cleavage of ECM proteins may facilitate VSMC migration based on the increased ADAMTS‐1 expression observed in atherosclerotic plaques. Our group highlighted the importance of ADAMTS‐5 in a mouse model of atherosclerosis 44: lower levels of ADAMTS‐5 accompanied accumulation of versican in aortas of apolipoprotein (apo)E‐knockout mice. Versican cleavage was barely detectable in aortas from ADAMTS‐5‐deficient mice providing conclusive evidence that in mice, ADAMTS‐5 is the key enzyme for versican degradation in the vasculature.

## Proteomic studies of the vascular ECM

**Figure 1:**
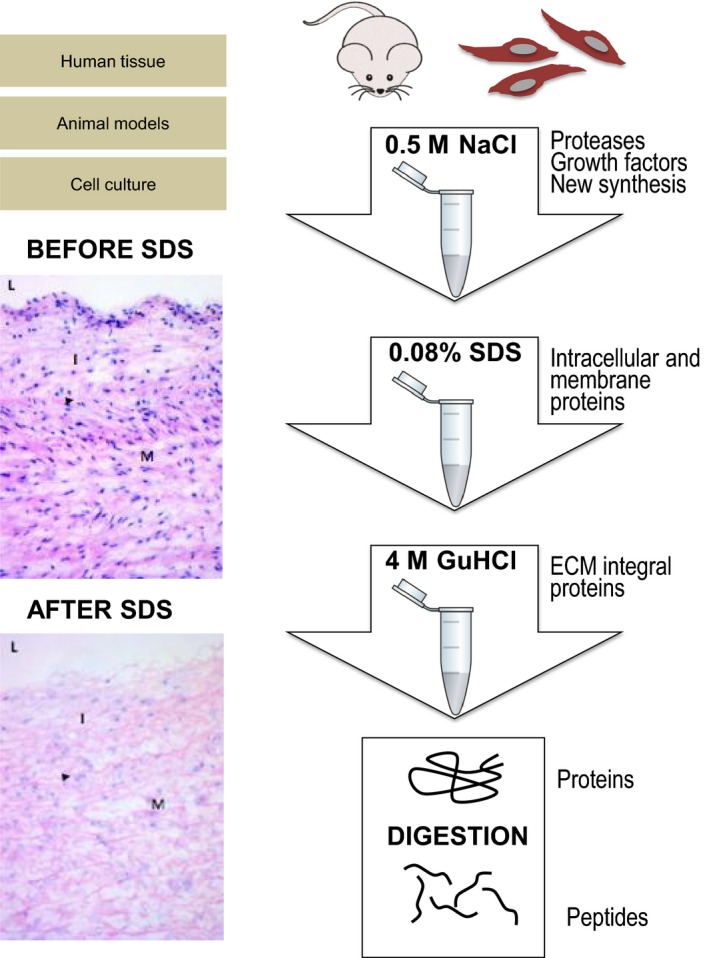
Proteomics workflow targeting the extracellular matrix (ECM). Biochemical subfractionation allows enrichment of ECM proteins. Vascular tissues of human origin or those obtained from animal models of cardiovascular disease are initially incubated in 0.5 mol L^−1^ NaCl to extract proteases, growth factors and newly synthesized ECM proteins. A subsequent decellularization step is performed with a buffer containing sodium dodecyl sulphate (SDS) before integral, polymeric ECM proteins are extracted with guanidine hydrochloride (GuHCl). Proteins obtained after sequential extraction are digested into peptides before liquid chromatography–tandem mass spectrometry analysis. The histological panel (haematoxylin and eosin staining) demonstrates the efficacy of SDS in removing cellular components before extraction of ECM proteins in human aortas. L, lumen; I, intima; M, media. Adapted from Didangelos *et al*. 18.

A comprehensive characterization of the ECM is essential for the understanding of vascular disease processes. Initially, the vascular ECM was mainly studied by antibody staining or by mRNA analysis. For example, Kirsch *et al*. 45 compared protein expression between human varicose and normal veins with antibodies against collagen IV, laminin, fibronectin and tenascin. Although the authors found significant differences between the diseased and healthy specimens, the study was limited to only a few ECM proteins. In cardiovascular research, proteomic studies predominantly investigate cellular proteins; thus, the extracellular space remains relatively underexplored. Even the ECM composition of different vascular territories is largely unknown. The detection of ECM proteins by proteomics was initially limited by problems in extracting insoluble ECM proteins and contamination from more abundant cytosolic and mitochondrial proteins. Since then, we and others have employed a stepwise extraction of ECM proteins that is amenable to analysis by liquid chromatography–tandem mass spectrometry (LC‐MS/MS) 18 (Fig. 1).

### Proteomics for the analysis of ECM composition

In one of a few studies that targeted the ECM, Talusan *et al*. 46 performed proteomic analysis on human pre‐atherosclerotic lesions from the carotid artery after PG extraction. Using internal thoracic arteries for a comparative control, they observed increased levels of lumican in the atherosclerotic‐prone carotid arteries. In the 10 years since this important work, no comprehensive proteomic analysis of the ECM has been performed in atherosclerosis. This is surprising, especially given the recent technological and methodological developments. Similar approaches have been utilized effectively in characterizing the composition of the ECMs of other normal and diseased tissues 47; in addition to our studies of the ECM signatures in abdominal aortic aneurysm 48 and aorta 18, Hansen and colleagues showed differential expression of a number of ECM proteins in the internal thoracic artery associated with arterial stiffness, as assessed by pulse wave velocity 49. The same group also found that levels of BM proteins were elevated in internal thoracic arteries of patients with type 2 diabetes mellitus 50.

There are few analogous studies, illustrating a significant void in the current literature (Table 1).

**Table 1:** Proteomics analysis after extracellular matrix (ECM) enrichment

Study/year	Tissue/species	Extraction method	Findings
Didangelos *et al*. (2010) 18	Aorta (Human)	0.5 mol L^−1^ NaCl (4 h) 0.08% SDS (4 h) 4 mol L^−1^ GuHCl (48 h)	321 total proteins 84 extracellular proteins
Naba *et al*. (2012) 81	Lung (Mouse)	Compartmental Protein Extraction kit	184 total proteins 55 matrisome proteins
de Castro Brás *et al*. (2013) 90	Left ventricle (Mouse)	0.5 mol L^−1^ NaCl (O/N) 1% SDS (3–5 days) 4 mol L^−1^ GuHCl (48 h)	157 total proteins 17 ECM proteins
Brachvogel *et al*. (2013) 91	Cartilage (Mouse)	1 mol L^−1^ NaCl (24 h) 4 mol L^−1^ GuHCl (24 h)	397 total proteins
Barallobre‐Barreiro *et al*. (2012) 92	Left ventricle (Pig)	0.5 mol L^−1^ NaCl (4 h) 0.08% SDS (4 h) 4 mol L^−1^ GuHCl (48 h)	139 extracellular proteins
Decaris *et al*. (2014) 93	Lung (Mouse)	0.5 mol L^−1^ NaCl (2 h) 0.08% SDS (16 h) 4 mol L^−1^ GuHCl (48 h)	N/A

### Proteomics for the analysis of PTMs of the vascular ECM

**Figure 2:**
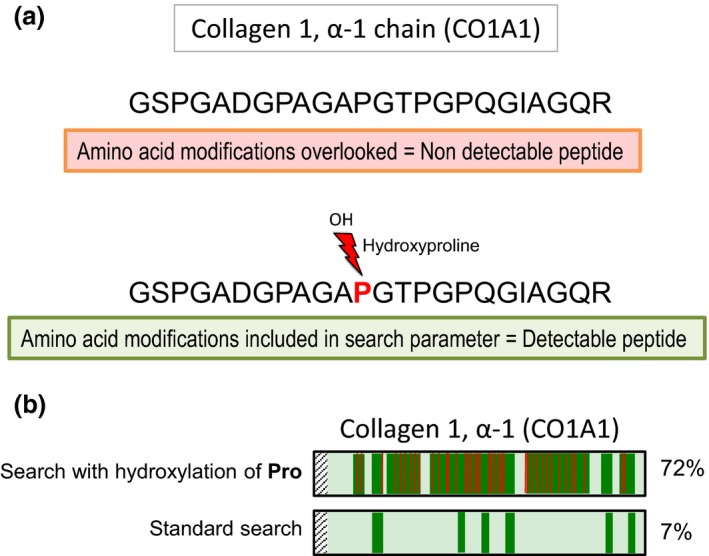
Proline hydroxylation of collagens. Proline hydroxylation (i.e. 4‐hydroxyproline and 3‐hydroxyproline) is a common modification that confers stability to collagen triple helices. (a) Inclusion of this variable modification in the search parameters dramatically improves identification and quantification of collagen peptides. The example shown is a peptide spanning amino acid positions 935–958 of human collagen 1, α‐1 chain (CO1A1). (b) CO1A1 protein sequence coverage is also improved ~10‐fold after searching liquid chromatography–tandem mass spectrometry data from human aortic specimen and including this variable PTM. Pro, proline.

**Figure 3:**
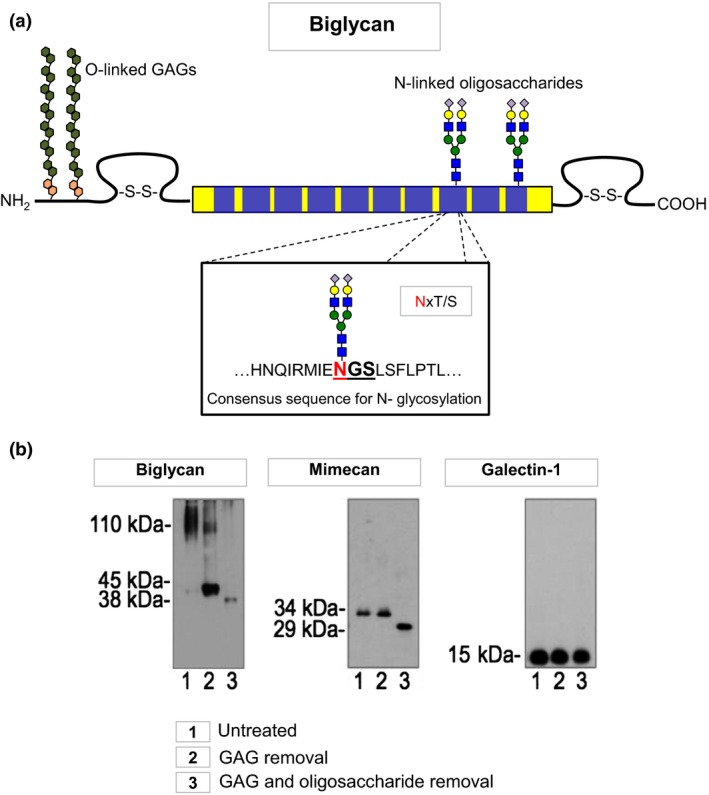
Glycosylation of extracellular matrix (ECM) proteins. More than 90% of ECM proteins are glycosylated, which can affect protein identification with antibodies. (a) Attachment of N‐linked oligosaccharides to ECM proteins occurs at specific consensus sequences. Biglycan is an example of a heavily glycosylated ECM protein. (b) Removal of glycosaminoglycans (GAGs) and small oligosaccharides or removal of GAGs alone affects protein migration by gel electrophoresis. Biglycan contains GAGs and oligosaccharides, and mimecan contains only N‐linked oligosaccharides, whilst galectin‐1 is a nonglycosylated ECM protein. The aim of glycoproteomics is the identification of the protein core and the attached glycan.

As discussed above, two PTMs are key for the analysis of the ECM: hydroxylation and glycosylation. The inclusion of hydroxylation of proline (i.e. a proline 16 Da heavier due to addition of OH^−^) in search parameters improves the detection of collagens (Fig. 2). Figure 3 shows the effect on selected ECM proteins (biglycan, mimecan and galectin‐1) of treating tissue extracts either with enzymes for GAG removal alone or a combination with five additional enzymes for the removal of complex oligosaccharides. Biglycan contains small oligosaccharide residues as well as larger GAGs. Few ECM proteins (e.g. galectin‐1) are devoid of glycosylations.

### Proteomics for the analysis of ECM degradation

As mentioned above, another important factor in ECM vessel wall remodelling is the role of MMPs. The earliest studies linking MMPs to vascular remodelling concerned intimal thickening following balloon angioplasty and vein grafting 51. Indeed, knockout studies in mice showed that MMP‐2, MMP‐9 and MMP‐14 were essential for neointimal hyperplasia. Consistent with these findings, *TIMP* gene transfer was also shown to hinder intima thickening in animal models of vascular balloon injury 52. However, despite showing promising signs as a potential therapeutic target, efforts to inhibit MMP activity using synthetic MMP inhibitors have yet to provide clinical benefits 53.

Seminal research by Henney *et al*. 54 implicated MMP‐3 in the human atherosclerotic plaque, leading a paradigm shift away from the concept that the atheroma is an inert obstruction. Since then, a spectrum of MMPs and TIMPs has been shown to be responsible for plaque destabilization including collagenases MMP‐1, MMP‐2 and MMP‐3 and TIMP‐1 and TIMP‐2. Much of our understanding of the role of MMPs in vascular diseases has come from transgenic or knockout mice with subsequent transcript and histological analysis; however, the results are often conflicting and confounding. For example, Luttun *et al*. 55 reported that MMP‐9‐null mice exhibited more stable plaques, compared to wild‐type animals as a result of a reduction in plaque size, macrophage content, collagen deposition and elastin degradation, whereas Johnson *et al*. 56 suggested that MMP‐9 was protective as the knockout model exhibited compositional changes associated with plaque instability.

We used proteomics to search for proteolytic fragments that were upregulated during ECM remodelling in abdominal aortic aneurysms 48. Using human samples, we showed that the degradation of six glycoproteins was linked to MMP‐12 activity. Similarly, we also used proteomics to identify potential substrates of other MMPs (MMP‐3, MMP‐9, MMP‐14) in vascular tissues 57. However, there is a need for more comprehensive analysis of the ‘degradome’ to investigate beyond the commonly studied MMPs.

### Targeted proteomics

A limitation of contemporary LC‐MS/MS is the bias towards highly abundant proteins. With large arteries and atherosclerotic vessels, this is exacerbated due to the higher cellular content and inflammatory cell infiltration in disease. Whilst enrichment steps have to a degree alleviated the problem, bias still remains. In an untargeted discovery proteomic experiment, the selection of peptides for fragmentation is a stochastic process leading to incompleteness of data and run‐to‐run variability. Instead, a targeted proteomic strategy can be employed that offers increased selectivity, sensitivity and quantitative accuracy. The most common targeted strategies are known as selected reaction monitoring and multiple reaction monitoring 58, 59. In essence, targeted proteomic analyses only consider the peptides of interest, as defined by the user. Thus, analysis time is not wasted on other peptides from highly abundant or contaminating proteins. Typically, targeted proteomics is utilized as a means to validate discovery proteomic data as the amino acid sequence information of the protein of interest must be known and the best peptides for targeted proteomic analysis should have already been identified. Targeted proteomics provides a method to quantify low‐abundance proteolytic fragments or protein isoforms 60, 61. This is particularly useful given that antibodies are not always available for neoepitopes generated after proteolytic cleavage of ECM proteins 57. Targeted proteomics is also applicable for studying PTMs.

### Glycoproteomics of the ECM

A common large‐scale proteomic strategy for N‐glycopeptide analysis utilizes a glycopeptide enrichment step followed by glycan removal. Typical methods to enrich glycosylated peptides involve the use of one or more of the following: lectins, hydrophilic interaction liquid chromatography, hydrazide or graphite 62. Subsequently, PNGase‐F is used to remove the glycan moiety, serving two purposes: first, the core peptide can be analysed without interference from sugars during fragmentation; and secondly, via a deamidation reaction, PNGase‐F converts the asparagine to aspartic acid. This conversion is characterized by a mass shift of 0.984 Da. If the reaction is performed in the presence of H_2_O^18^ (water that contains an isotopic form of oxygen: oxygen‐18 instead of oxygen‐16), this leads to a mass shift of 2.98 Da 63 and further improves confidence that the site was glycosylated and the conversion did not occur spontaneously. We have previously treated proteins secreted by endothelial cells with PNGase‐F in the presence of O^18^‐labelled water prior to LC‐MS/MS analysis 64. More recently, we applied the same method to more complex extracts from human vascular tissues. Treatment with PNGase‐F in O^18^‐labelled water allowed the identification of peptides that contain glycosylated asparagines, leading to improved sequence coverage for heavily N‐glycosylated ECM proteins such as the SLRPs decorin, biglycan and lumican, or the BM‐related glycoprotein fibronectin. The combined analysis of the glycan (glycomics) 65, 66 and the protein (proteomics) forms the field of glycoproteomics. Enzymatically deglycosylating a glycoprotein allows for the separate analysis of the core protein and of the glycan 67, but the aim of glycoproteomics is to combine both analyses, which makes it a more challenging task: first, all proteins in the sample are digested into peptides, and then, the glycopeptides can be enriched followed by analysis by LC‐MS/MS. The obtained fragmentation spectrum can be very complex, because it contains signals from the peptide backbone, the sugar moiety and a combination of both (peptide fragments with the sugar still attached) 68. The interpretation of such spectra requires significant experience in interpretation, manual assignment and evaluation. Consequently, high‐throughput analysis is precluded at present. Nevertheless, glycoproteomics can be used to systematically profile a given proteome 69.

## Retention of lipoproteins in the vasculature

Further motivation for proteomic studies of the vascular ECM arises from our lack of understanding with regard to the ‘response‐to‐retention hypothesis’ in early atherosclerosis 70. Evidence from biochemical and molecular biology studies now supports the theory that ECM proteins, in particular vascular PGs, are involved in the retention of lipoproteins. However, it is still not clear why some vessels are more susceptible to atherosclerosis than others. This disparity is likely to reflect inherent variations in vascular properties and may include differences in ECM composition in response to wall stress and shear flow that warrant proteomic investigation.

Progression of atherosclerosis involves the formation of a lipid‐rich necrotic core, and, as part of the inherent repair process that ensues, a fibrous cap forms over the core 71. The ECM constituents of this core along with the VSMC content have been inextricably linked to the stability of the plaque 72. Collagen I accounts for ~60% of the total protein in the fibrous cap, as corroborated by gene expression data from atherosclerotic lesions in mice 73. In fact, the most over‐expressed genes associated with intimal hyperplasia encode ECM proteins such as collagens I and III, in addition to PGs. Given the tensile strength of fibrillar collagens, it is perhaps unsurprising that their presence is associated with stable plaques. By contrast, vulnerable plaques are characterized by a thin cap and a reduction in fibrillar collagen. Intimal PGs are upregulated at sites of intimal thickening and contribute to the development of atherosclerosis by sequestering apoB‐containing lipoproteins which in turn stimulate the canonical chronic inflammatory response in the vessel wall. Thus, we have endeavoured to characterize the lipid content of vulnerable and stable plaques 74 and identify lipidomic signatures for CVD 75.

Given the vascular retention and accumulation of apoB‐containing lipoproteins, the importance of remnants from triglyceride‐rich lipoproteins has recently been recognized. Evidence from Mendelian randomized studies now suggests a primary role of triglyceride‐rich lipoproteins in CVD 76, 77. The lipolysis of triglycerides results in the conversion of chylomicrons into chylomicron remnants and very low‐density lipoprotein (VLDL) particles into intermediate‐density lipoprotein (IDL) and low‐density lipoprotein (LDL). Lipoprotein lipase (attached to the endothelium of blood capillaries) mediates this process by hydrolysing the triglycerides carried in chylomicrons and VLDL. Besides LDL, the lipolysis of triglycerides yields triglyceride‐depleted but cholesterol‐rich remnants (chylomicron remnants, VLDL, and IDL in the nonfasting state, and VLDL and IDL in the fasting state). The underlying mechanism of remnant atherogenicity is thought to be similar to that of LDL; that is, the triglyceride‐depleted and cholesterol‐rich remnant particles are trapped by the connective tissue in the arterial wall. Chylomicrons and VLDL particles are too large to penetrate the arterial wall. Remnants, however, are small enough and may act in the same atherogenic way as LDL. Of interest, Varbo *et al*. provided evidence to suggest that remnants are causally associated with inflammation and ischaemic heart disease, whereas LDL was found to have only a causal relationship with ischaemic heart disease, indicating that the inflammatory component in atherosclerosis is predominantly driven by remnant particles 78. Furthermore, it has been suggested that nonfasting remnant cholesterol levels may serve as a better biomarker to predict CVD 79. Ultimately, a comprehensive analysis of molecular lipid species (lipidomics) and apolipoproteins is required in CVD (see review by Hinterwirth *et al*. 80).

## Other translational applications to vascular disorders

### ECM proteins as biomarkers

Pursuing efforts to characterize ECM composition has potential beyond the description of ECM remodelling at a molecular level. Naba *et al*. 81 have shown that comparing the extracellular proteome of normal and cancerous tissues can lead to biomarker identification. More importantly, de Kleijn *et al*. 82 found that the level of the ECM glycoprotein osteopontin in atherosclerotic plaques was predictive for CVD events. As described above, MMPs and TIMPs are currently believed to be the major proteolytic mediators in the vessel wall. Instead of circulating MMPs and TIMPs 83, the proteolytic fragments of ECM proteins could be used as measures of disease progression. This possibility is particularly appealing because of the release of these fragments into body fluids. For instance, serological levels of elastin peptides and the amino terminal of propeptide of collagen III (PIIINP) have been correlated with abdominal aneurysm progression 84. PIIINP has also received attention as a marker of atherosclerosis 85. Thus, it is likely that a proteomics approach targeting the ECM will reveal new biomarker candidates with vascular specificity. ECM biomarkers of clinical interest are summarized in Table 2.

**Table 2:** Extracellular matrix biomarkers in cardiovascular disease (CVD)

Biomarker	Tissue	Clinical context	Main findings	Reference
Biglycan	Coronary artery	Atherosclerosis	Atherosclerotic segments have enriched deposits of biglycan	94
Decorin	Coronary artery	Atherosclerosis	Accumulation in intima of early atherosclerotic lesions	95
Podocan	Coronary artery	Restenosis	Reduced in restenotic coronary lesions	96
Fibromodulin	Coronary artery	Atherosclerosis	High expression associated with symptomatic plaques	97
MMP‐1	Serum	Atherosclerosis	Serum levels associated with plaque burden	98
MMP‐9	Plasma	CVD	Plasma concentration predictor of cardiovascular mortality	99
MMP‐10	Serum	PAD	Increased levels associated with clinical events	100
MMP‐12	Plasma	Atherosclerosis, CAD	Associated with severe atherosclerosis and increased incidence of coronary events	101
TIMP‐1	Plasma	CVD	Predictive of all‐cause death, MI and cardiac mortality	102
ADAMTS‐1	Aorta and coronary artery	Atherosclerosis	Upregulated in the intima when plaque is present	43
Cathepsin G	Plasma	Atherosclerosis	Reduced in patients with atherosclerosis	103
Cathepsin K	Plasma	CAD	Elevated plasma levels in patients with CAD	104
Collagen III	Serum	Acute MI	High levels of collagen III fragments predict mortality	105
	Serum	CAD	Collagen III fragments predictive for atherosclerosis	85
Collagen IV	Coronary artery	Atherosclerosis	Aldehyde modifications in collagen IV increase risk of clinical events	106
Osteopontin	Carotid artery	Atherosclerosis	Predictive of vascular complications	82
Periostin	Cardiac valve	Atherosclerotic VHD	Upregulated in patients with atherosclerotic VHD	107
Versican	Plasma	Atherosclerosis	Degradation fragments elevated in patients with atherosclerotic diseases	108
Fibronectin	Abdominal aorta	Atherosclerosis	Enriched in atherosclerotic plaques	109
Elastin	Carotid artery	Atherosclerosis	Elevated levels of elastin fragments associated with symptomatic carotid stenosis	110
Tenascin‐C	n/a	Atherosclerosis	SNP associated with atherosclerosis and CAD	111
Matrix Gla protein	n/a	Atherosclerosis	SNP influences calcification and is associated with increased risk of MI	112
Galectin‐3	Plasma	CAD	Increased levels are strong predictor of cardiovascular death	113

CAD, coronary artery disease; CVD, cardiovascular disease; MI, myocardial infarction; PAD, peripheral artery disease; SNP, single nucleotide polymorphism; VHD, valvular heart disease.

### ECM proteomics in drug discovery

Despite the number of diseases involving the ECM, there are currently few drugs that specifically target ECM components. The most prominent examples of such drugs to date are those that inhibit integrins, such as abciximab (to treat thrombosis) and natalizumab (to treat multiple sclerosis and Crohn's disease) 86. However, there are numerous examples of other drug classes that indirectly influence the ECM in a favourable manner. For instance, it has been postulated that angiotensin II antagonists also act on the ECM by inhibiting TGFβ 87. Furthermore, in patients with Marfan's syndrome, these drugs were found to slow the rate of progression of aortic‐root dilatation. Unfortunately, these concomitant effects on the ECM are largely nonspecific. Despite successful precedents, it has been noted that ECM proteins are mainly overlooked in drug discovery efforts 4.

## Conclusions

During the progression of many vascular disorders, the continuous pathological remodelling of the ECM contributes to the manifestation of the disease; for instance, the transition of a stable to a vulnerable lesion in atherosclerosis 83, the degradation and subsequent wall thinning in abdominal aortic aneurysm 48 and neointimal hyperplasia in vein graft disease 88 and varicose veins 89. The increasing performance of proteomic platforms and the ability to provide large proteomic datasets, coupled with system biology approaches, will ultimately facilitate the identification of novel ‘druggable’ targets for and biomarkers of CVD.

## Conflict of interest statement

None.
